# The ‘two toe’ technique for femorofemoral bypass

**DOI:** 10.1308/rcsann.2014.96.1.79a

**Published:** 2014-01

**Authors:** A Thapar, S Dindyal, J Refson

**Affiliations:** Princess Alexandra Hospital NHS Trust,UK

During a femorofemoral bypass, the heel of a dacron graft may be compressed under the inguinal ligament, with the subsequent risk of occlusion. Furthermore, an additional patch may be required to close larger arteriotomies. The following technique addresses both these difficulties without incising the inguinal ligament. The graft is split into two ‘toes’, instead of a ‘heel’ and a ‘toe’. The inguinal ligament now lies over the superior toe of the graft instead of compressing the heel of the graft. The toes can be lengthened if the arteriotomy is extended into the profunda femoris, avoiding the need for a patch.

**Figure 1:**
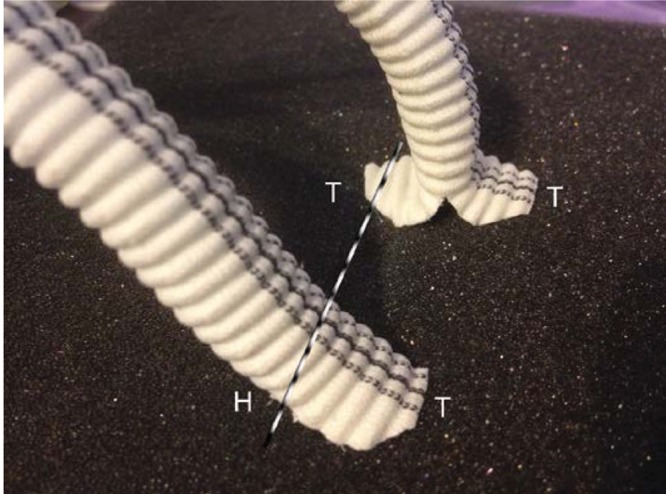
The ‘two toe’ technique (background) is shown side by side with the conventional ‘cobra hood’ (foreground). The dotted line represents the inguinal ligament, which compresses the main body of the conventional graft but not the two toe graft

